# Merkel Cell Carcinoma: An Otolaryngological Point of View of An Unusual Sinonasal Mass

**DOI:** 10.7759/cureus.31676

**Published:** 2022-11-19

**Authors:** André De Sousa Machado, Ana Silva, Jose Brandao, Luis Meireles

**Affiliations:** 1 Otolaryngology, Centro Hospitalar Universitário do Porto, Porto, PRT; 2 Pathology, Centro Hospitalar Universitário do Porto, Porto, PRT

**Keywords:** nose surgery, dermato-oncology, nasal neoplasms, ear nose and throat, merkel cell cancer

## Abstract

Merkel cell carcinoma is a pathologic diagnosis mainly observed in sun-exposed cutaneous areas, like the head and neck. Ultraviolet (UV) exposure and immunosuppression are the common predisposing factors. Merkel cell carcinoma of the head and neck is quite an uncommon disease. This case report involves a 56-year-old man who exhibited a skin lesion on the nasal dorsum with a mass in the right maxillary sinus. The biopsies from both sides were diagnostic for Merkel cell carcinoma. The patient underwent endoscopic sinus surgery and removal of the skin lesion with free margins. The patient has been free of disease for the last 20 months now and maintains follow-up with endoscopy and imaging in the Ear Nose Throat office. Only a few cases of Merkel cell carcinoma of the nasal mucosa have been reported in the literature. We report our approach and management of this rare pathologic presentation.

## Introduction

Merkel cells are neuroendocrine cells with electron-dense neurosecretory granules and neurofilaments in the cytoplasm of their cells, hence Merkel cell carcinoma (MCC) is considered a neuroendocrine tumor. Neuroendocrine markers like synaptophysin and chromogranin are expressed in MCC, which is a neuroendocrine carcinoma. As opposed to small cell carcinoma of the lung, MCC is usually negative for TTF-1. A perinuclear pattern of CK20 is observed in MCC, which is known to express keratins. MCC has also been associated with Merkel cell polyomavirus, which can be detected using immunohistochemistry (IHC) or polymerase chain reaction (PCR) [1,2]. Despite being the second-most common cause of skin neuroendocrine cancer, MCC is a rare entity and presents with an aggressive course, frequently with intravascular invasion [1-3]. Despite surgical and medical treatment, a high degree of local recurrence and distant metastasis is common in this disease, resulting in high mortality [1-3]. Several factors pose a high risk for MCC occurrence, such as heavy and unprotected ultraviolet radiation exposure and Merkel cell polyomavirus infection. Also, the use of immunosuppressive medication for an extended period is associated with the occurrence of MCC [4]. MCC involving the nasal mucosa is exceedingly rare, and only a few case reports and small case series of MCC occurring in nasal mucosa have been reported [5-12].

## Case presentation

**Figure 1:**
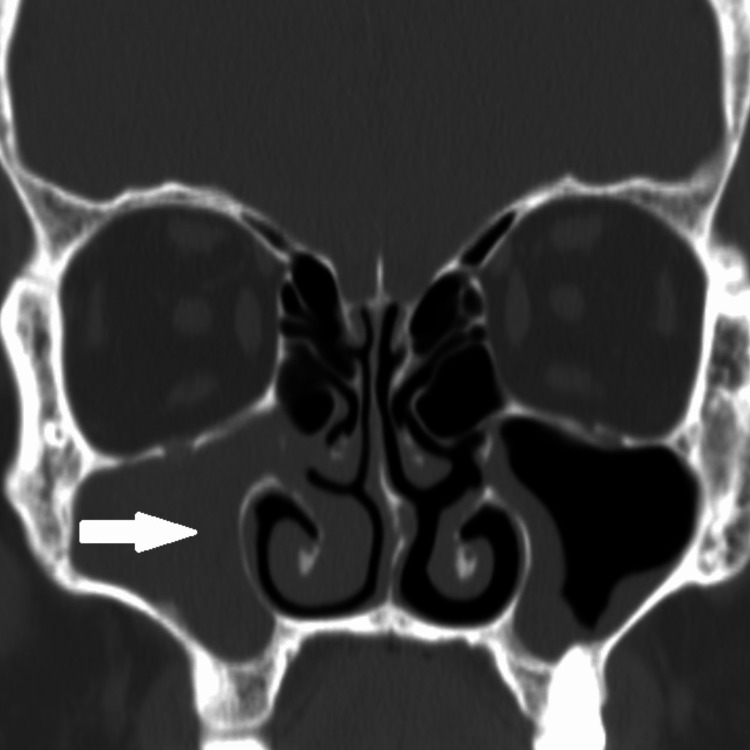
Pre-operative coronal bone window CT image at the level of maxillary sinus showing soft tissue density filling the right maxillary sinus; the bony contours of the sinus remain intact (white arrow).

**Figure 2:**
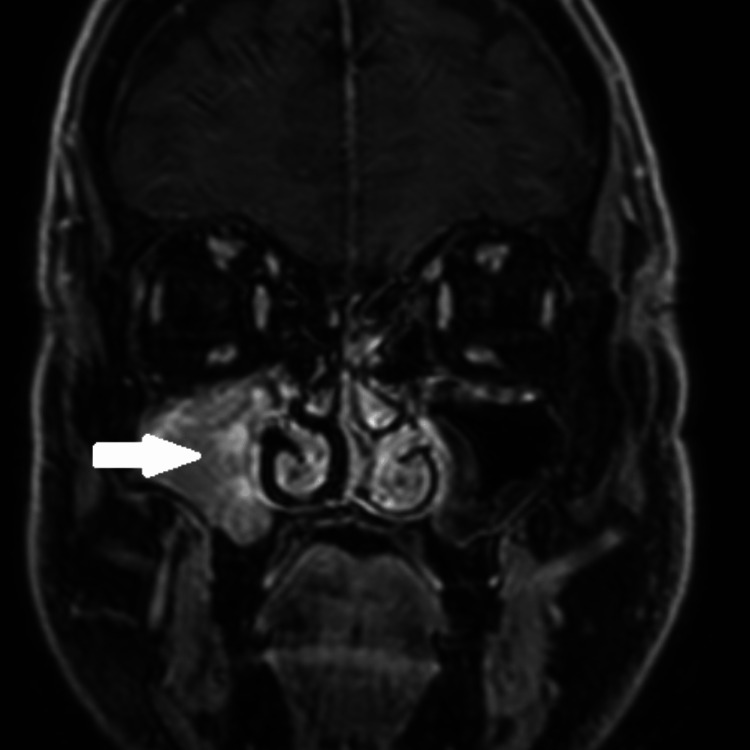
Pre-operative coronal contrast-enhanced MRI-T1 fat-saturated image at the level of maxillary sinus showing the same lesion restricted to the interior of the right sinus, with avid and slightly heterogeneous enhancement (white arrow).

A 56-year-old man presented with a skin lesion of two-month evolution on the nose with progressive enlargement and complaints of unilateral nasal blockage. No other nasal symptoms were described by the patient. The skin lesion on the nasal dorsum was painless, hard, and hyperpigmented. The symptoms were first addressed in primary care with topic corticosteroids and after, the patient was referred to the otolaryngology clinic for further evaluation. A rigid nasal endoscopy with 0, 30, and 70 degrees was performed with no visualization of masses. A CT scan and MRI revealed a lesion restricted to the interior wall of the right maxillary sinus with soft tissue density (Figures 1-2). Nasal endoscopy revealed a fleshy mass arising above the osteomeatal complex with no ulceration or signs of bony erosion.

The bony walls were intact. A few weeks later, the patient presented with a skin lesion in the nasal dorsum de novo with the same features previously presented. After a new biopsy of the lesion, a Merkel carcinoma (positive for synaptophysin, chromogranin, CK20, CAM 5.2, and Merkel cell polyomavirus). The nasal mass biopsy revealed MCC with surrounding normal sinonasal submucosa. Immunostains showed the tumor cells were positive for synaptophysin, chromogranin, CK20, CAM 5.2, and Merkel cell polyomavirus, and a dot-like peri-nuclear pattern was seen in the same. Positron emission tomography (PET) also showed a hypermetabolic right maxillary sinus and nasal dorsum with high-density content. The abdominal and pelvic CT did not show any suspicious lesions. The patient's case was discussed in a multidisciplinary team meeting, and the team decided on surgical treatment.

**Figure 3:**
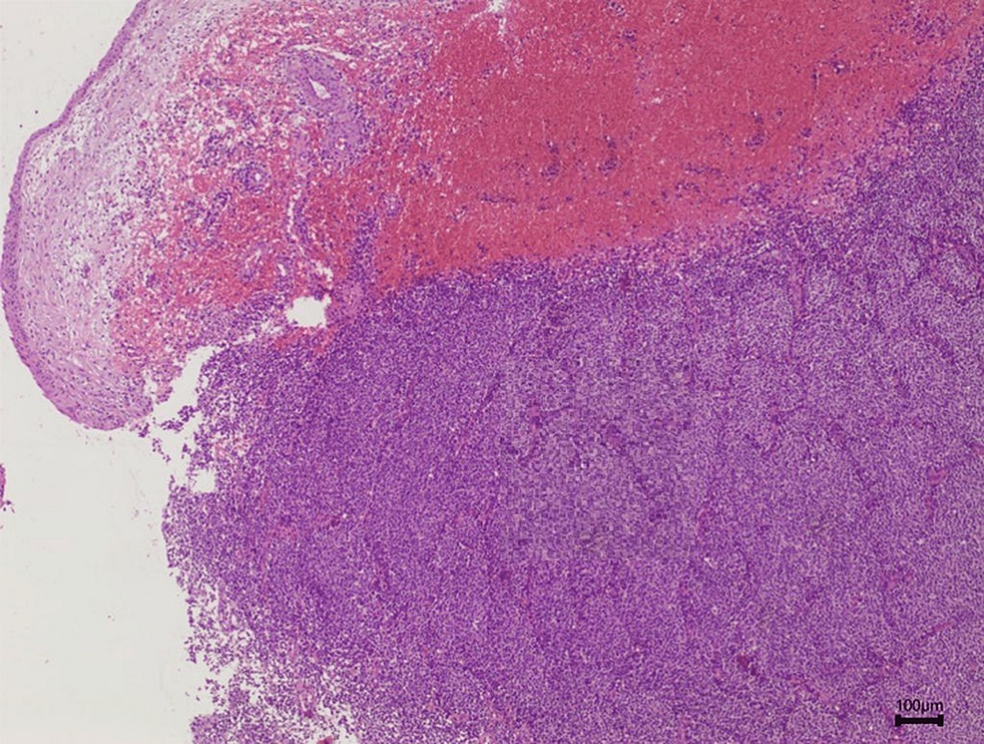
Pseudostratified respiratory epithelium lined with tumor tissue. Magnification 40x

**Figure 4:**
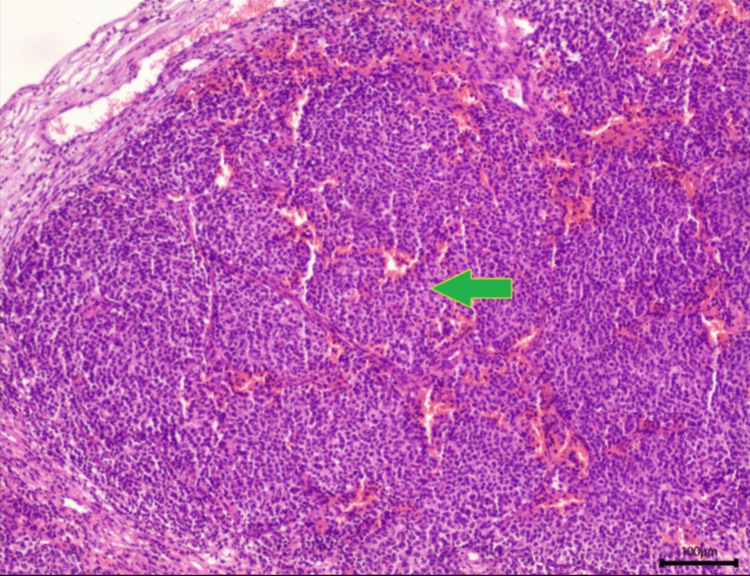
Tumor tissue with atypia and metaplasia compatible with Merkel cell carcinoma (green arrow). Magnification 40x

**Figure 5:**
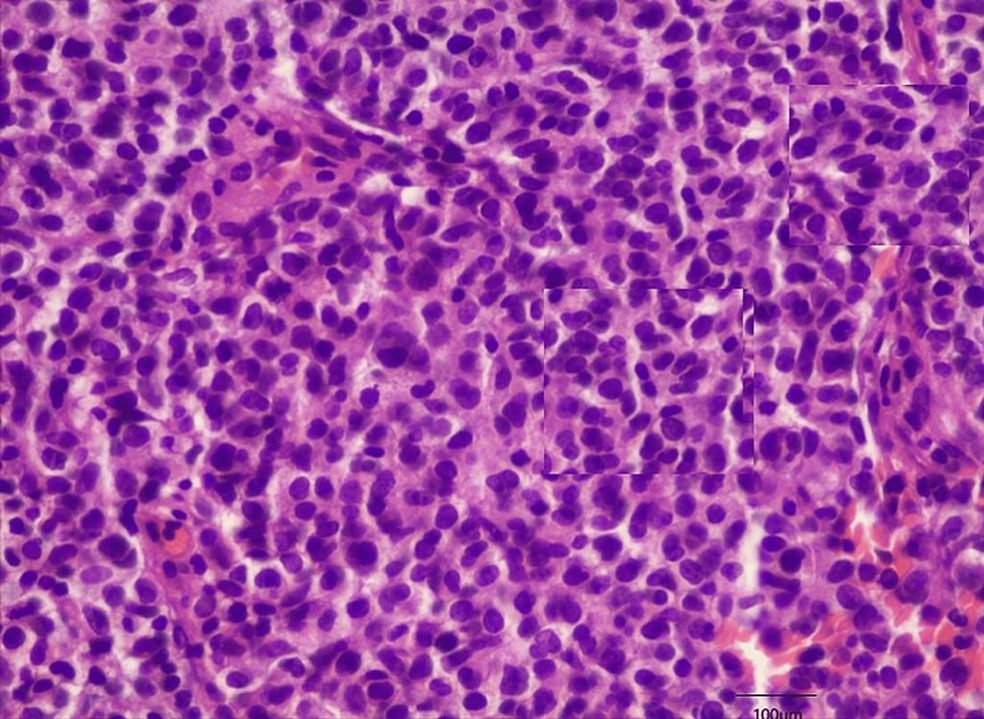
Cells with atypia and metaplasia compatible with Merkel cell carcinoma. Magnification 100x

**Figure 6:**
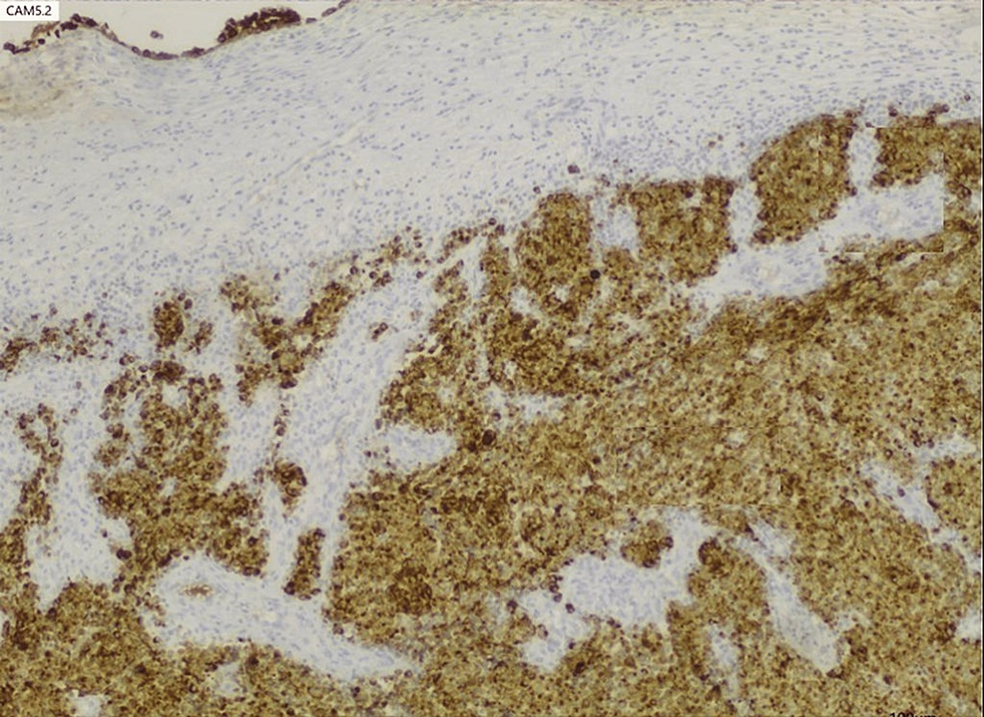
Positivity of the sample for CAM5.2. Magnification 40x

**Figure 7:**
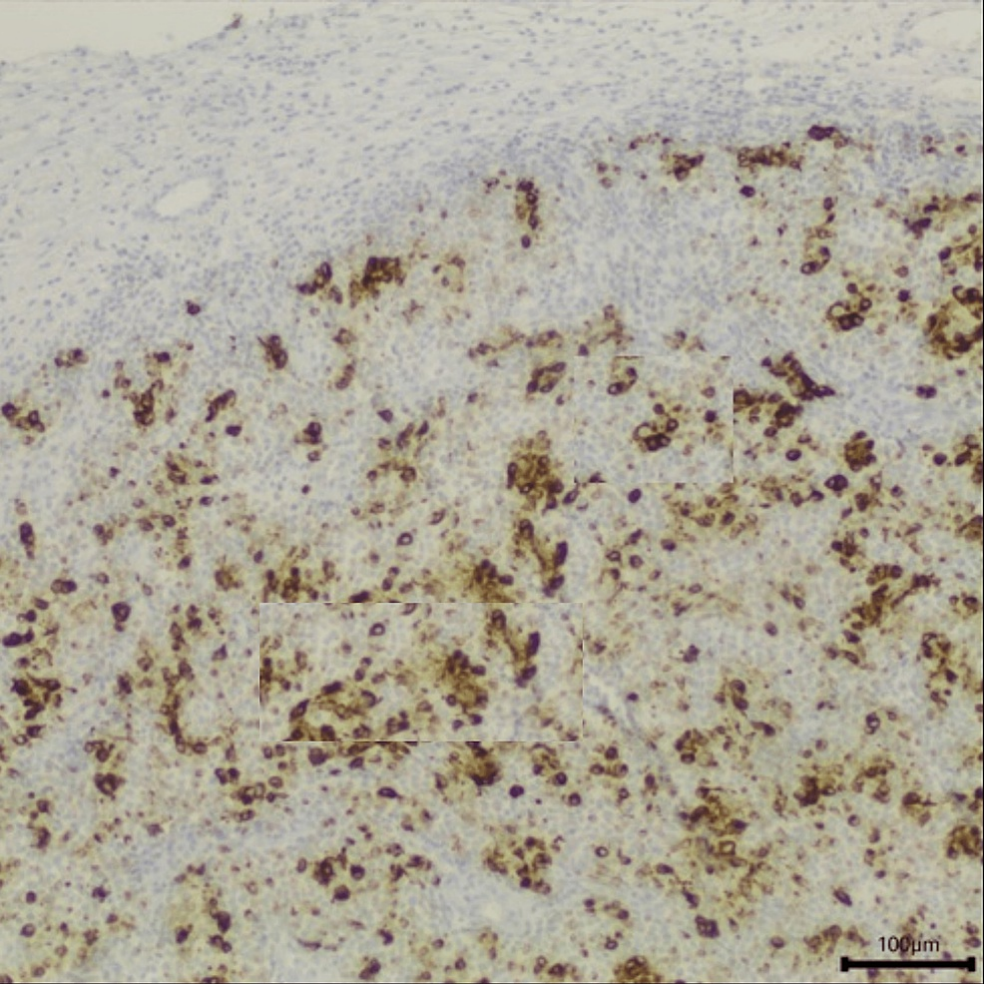
Anatomopathological study of the sample collected in endoscopic sinus surgery with positivity for CK20. Magnification 100x

**Figure 8:**
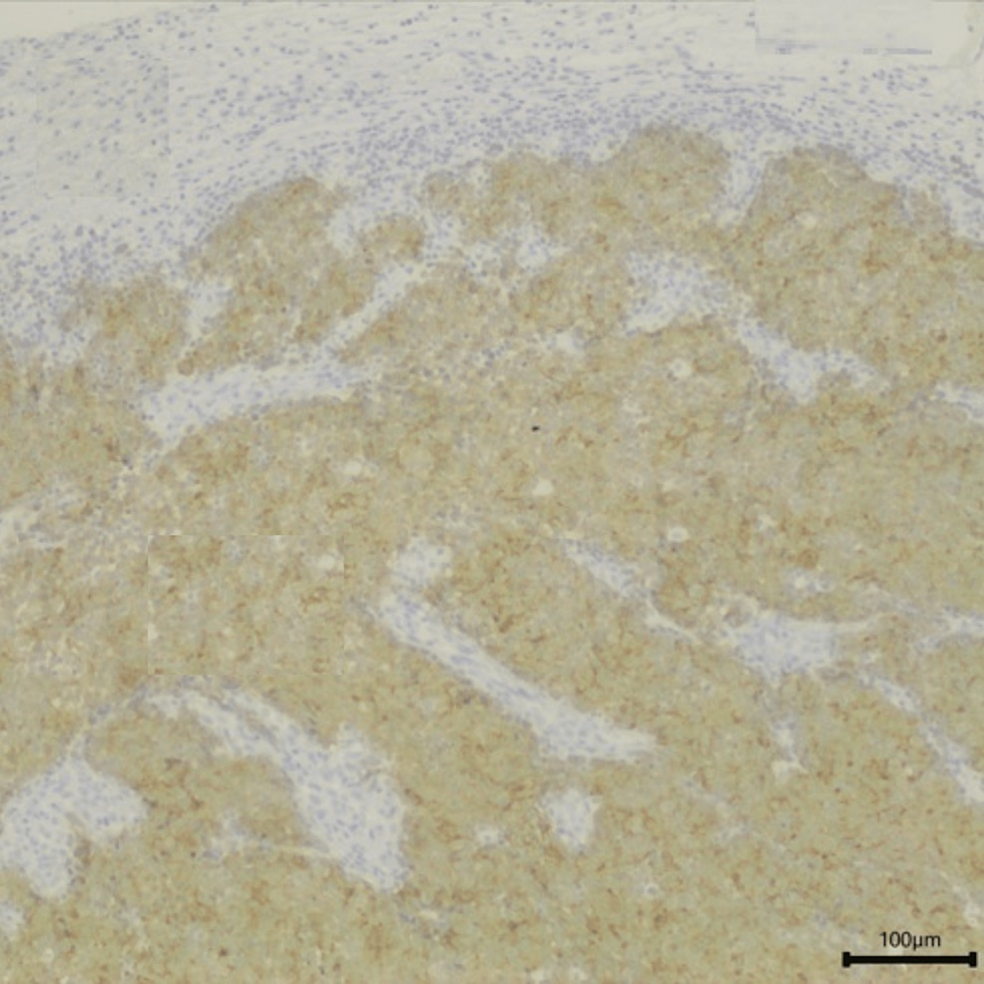
Anatomopathological study of the sample collected with positivity for synaptophysin (black arrow). Magnification 100x

**Figure 9:**
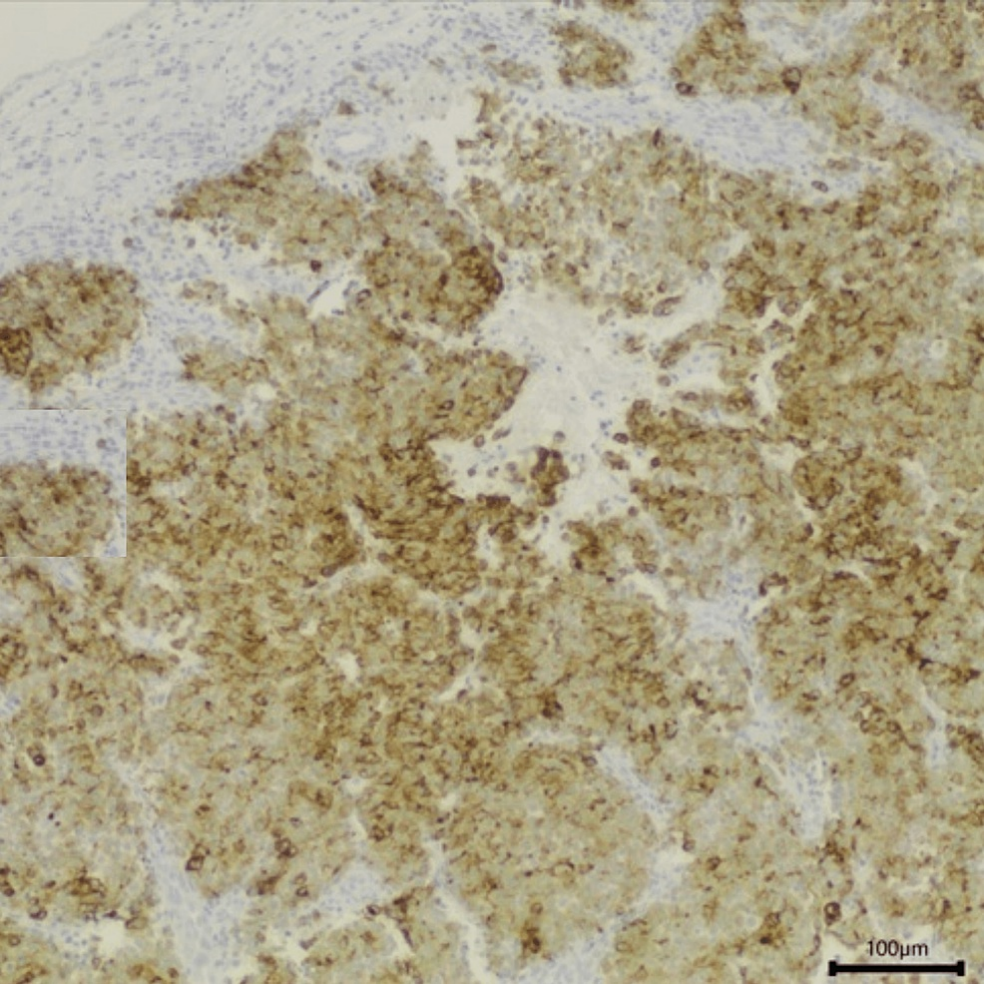
Histology of the sample collected with positivity for chromogranin A. Magnification 100x

The skin lesion was excised with free margins and was revealed to be MCC. Endoscopic sinus surgery was performed to resect the lesion and establish the diagnosis. The surgery consisted of right endonasal uncinectomy, ipsilateral maxillary sinusotomy with medial maxillectomy, and endonasal excision of the lesion. No perioperative or postoperative complications were observed. The anatomopathological study revealed a Merkel carcinoma positive for synaptophysin, chromogranin, CK20, and Merkel cell polyomavirus (Figures 3-9).

**Figure 10:**
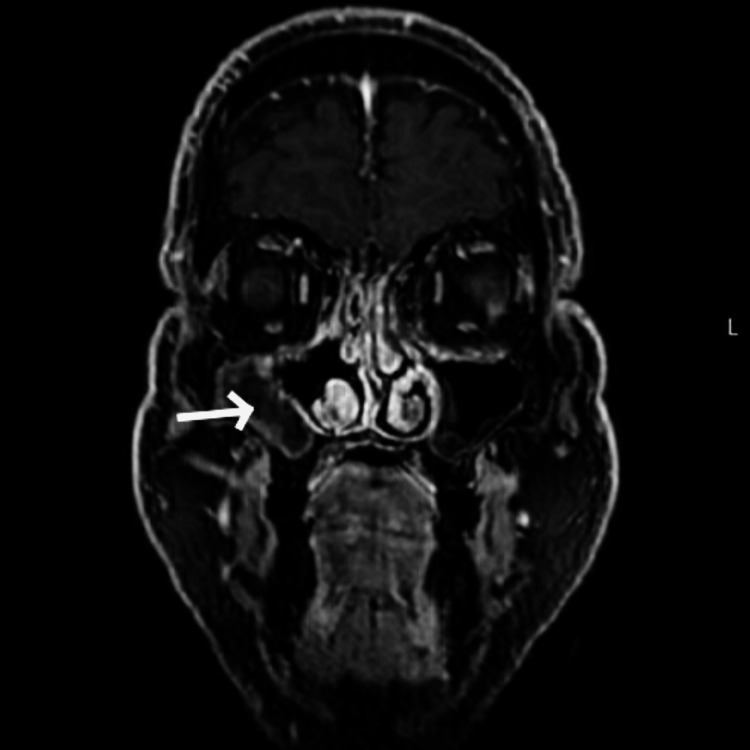
MRI after endoscopic sinus surgery of right maxillary sinus (white arrow). Coronal contrast-enhanced MRI T1 fat-saturated image at the level of maxillary sinus after surgical treatment, showing middle meatal antrostomy and removal of the enhancing lesion, with post-surgical findings with no signs of residual lesions (white arrow). Right-handed side.

As a complementary treatment, the patient underwent bilateral supraomohyoid neck dissection with adjuvant radiotherapy (VMAT-SIB technique) toward the nose, right maxillary sinus, and lymph nodes in levels I, II, and III. Clinical tolerance was observed, with only mild xerostomia and odynophagia being highlighted. The presence of nasal mucositis was only observed during and after radiotherapy. The patient remains under follow-up for 30 months. MRI scans of the nasal and paranasal sinus, nasal endoscopy, and cervicothoracic CT show no evidence of locoregional recurrence (Figure 10).

## Discussion

MCC is a rare cutaneous carcinoma first described by Toker as “trabecular carcinoma” [6]. Often seen in elderly patients with a history of extensive sun exposure, MCC most commonly occurs in the head and neck [13,14]. Most tumors have already metastasized with a preference for regional lymph nodes by the time the tumor is diagnosed [15].

We report a case of a skin MCC metastasizing to the maxillary sinus without extension into the nasal cavity - confirmed by the continuity of the lesion and by histopathological analysis - and without detectable metastasization into regional lymph nodes. As far as we know, there are few cases of MCC in the sinuses or nasopharynx [5-11]. The differential diagnosis of neuroendocrine carcinomas in the sinus is extensive and challenging: olfactory neuroblastoma (positive for synaptophysin and chromogranin; negative for cytokeratins), small and large cell neuroendocrine carcinomas (negative for CK20 and Merkel cell polyomavirus), and sinonasal undifferentiated carcinoma (SNUC) (positive for cytokeratins; negative for CK20 and Merkel cell polyomavirus) [15-17]. Merkel cell polyomavirus causes this aggressive and rare cancer and a relatively innocuous infection. When comparing the overall survival of nasal MCC without metastasis, our findings are consistent with the literature. While metastasis to the thyroid is more frequently seen, this was not found in our case [8,10]. We recommend otolaryngology follow-up every six years with nasal endoscopy, MRI, and cervical palpation [18]. 

The recommended workup for nasal MCC and treatment is still a theme of debate and no consensus was found in the literature, although a combination of surgery and radiotherapy is advocated, and followed by our team [19]. Early diagnosis is of the utmost importance to patients, as it can improve their prognosis; however, the rarity of these tumors, misinterpretation of small round blue cell tumors in the skin, and lack of a proper clinical history can compromise effective treatment, contributing to a disease-specific survival rate between 30% and 64%. This survival rate applies to MCC in general; due to the scarcity of data related to nasal MCC the survival rate is still not known and is a theme of debate [19]. It is estimated that 56% of MCCs are benign at biopsy [20].

## Conclusions

Merkel cell carcinoma is most commonly seen within the skin in areas with heavy sun exposure, mainly the head and neck, with relation to Merkel cell polyomavirus infection. It is uncommon for Merkel cell carcinoma to appear at extracutaneous mucosal sites of the head and neck. In addition to Merkel cell polyomavirus testing, it is important to consider Merkel cell carcinoma in extracutaneous locations. Early diagnosis is extremely important to improve the prognosis and outcomes of these patients.
